# Sensitivity of resting magnetic resonance first-pass myocardial perfusion imaging for the detection of acute and chronic myocardial infarction

**DOI:** 10.1186/1532-429X-11-S1-P156

**Published:** 2009-01-28

**Authors:** Alejandro Aquino, Edwin Wu, Thomas A Holly, Daniel C Lee

**Affiliations:** Northwestern University, 633 Clark Street Evanston, Chicago, 60208 IL USA

**Keywords:** Cardiac Magnetic Resonance, Myocardial Blood Flow, Viable Myocardium, Perfusion Study, Acute Infarct

## Background

At rest, myocardial blood flow in an area of myocardial infarction is roughly 20% that of blood flow in remote myocardium [1]. Since magnetic resonance first-pass myocardial perfusion imaging has been shown to effectively identify 2:1 differences in vasodilated blood flow in viable myocardium [2], one would expect that the 5:1 difference in resting blood flow between infarcted and viable myocardium would be easily identified. However, the ability of resting first-pass perfusion imaging to detect areas of infarcted myocardium is unknown.

## Objective

The purpose of this study was to determine the sensitivity of resting cardiac magnetic resonance (CMR) perfusion for evaluating the presence and extent of myocardial infarction, as defined by delayed contrast-enhanced (DE) CMR.

## Methods

59 patients were imaged serially following acute ST-segment elevation myocardial infarction (MI) by CMR cine and DE as part of a study evaluating the ability of CMR indices to predict post-MI remodeling [3]. We retrospectively identified 83 studies in which resting CMR perfusion was performed within the same examination as DE. Short axis perfusion and DE images were obtained within 7 days (30 acute studies) and more than 3 months (53 chronic studies) following MI. CMR studies were visually analyzed by two blinded readers on a 16-segment model. Each segment was scored independently for the presence and extent of DE (0 = 0%, 1 = 1–25%, 2 = 26–50%, 3 = 51–75%, 4 = 76–100%) and resting perfusion defect (0 = no defect, 1 = mild defect, 2 = moderate defect, 3 = severe defect). For sensitivity analysis, DE was considered the gold standard for presence of MI.

## Results

**Figure 1:**
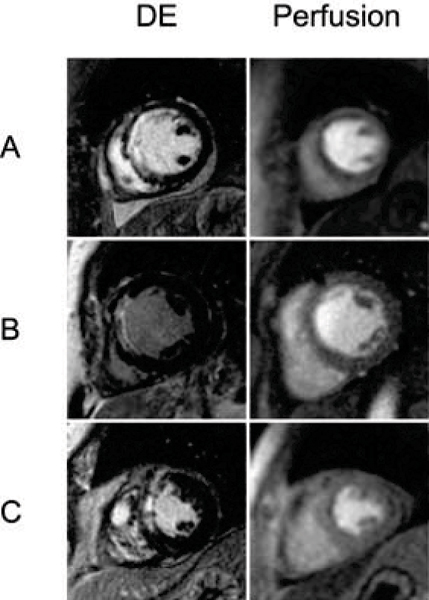
**Panel A depicts a patient in whom the size and extent of perfusion defect and delayed contrast enhancement (DE) are matched**. In Panel B, no perfusion defect is seen despite a large area of DE. In Panel C, the perfusion defect significantly underestimates the size of DE.

For all studies with MI identified by DE, the sensitivity of resting CMR perfusion for the detection of MI was 71% (59/83). The sensitivity was higher in patients with acute infarcts (26/30, 87%) than for patients with chronic infarcts (33/53, 62%). The mean number of segments demonstrating hyperenhancement was higher than the mean number of segments demonstrating resting perfusion defects (4.7 vs 2.5, p < 0.0001). When comparing patients with true-positive perfusion studies to those with false-negative perfusion studies, the mean hyperenhancement score was higher (10.6 vs 6.0, p < 0.001). Figure 1.

## Conclusion

The sensitivity of resting magnetic resonance first-pass myocardial perfusion imaging for the detection of myocardial infarction is relatively low, and the extent of hyperenhancement is larger than the extent of perfusion defects. Sensitivity is higher for acute infarcts than for chronic infarcts, and smaller infarcts are more likely to be missed. When performing first-pass perfusion studies, delayed contrast-enhanced imaging also needs to be performed for accurate assessment of the size and extent of myocardial infarction.
